# A successful treatment of necrotizing fasciitis following the surgery of distal radius plate removal: A case report and literature review

**DOI:** 10.1097/MD.0000000000010305

**Published:** 2018-04-13

**Authors:** Yuchen Cai, Yaokai Gan, Chao Yu, Jian Tang, Yuehua Sun

**Affiliations:** Department of Orthopaedic Surgery, Shanghai Ninth People's Hospital Affiliated to Shanghai Jiao Tong University School of Medicine, Shanghai, China.

**Keywords:** bone plate removal surgery, infection, necrotizing fasciitis, therapeutics

## Abstract

**Rationale:**

Necrotizing fasciitis (NF) is defined as a rare, rapidly progressive, and highly lethal skin infection characterized by necrosis of the fascia and subcutaneous tissue.

**Patient concerns:**

The present study aims to discuss the case of a 35-year-old man who developed NF following a routine sterile right distal radius bone plate removal surgery.

**Diagnoses:**

The patient was suspected of NF based on his clinical manifestations, laboratory tests, and imaging results. The diagnosis of NF was confirmed by histological examinations.

**Interventions:**

Serial prompt and extensive debridement was performed during the rapid and aggressive extension of the skin infection, together with antibiotics and supportive treatments.

**Outcomes:**

The condition of the patient finally improved on the sixth day of disease progression. Skin grafting of his right forearm wound was performed successfully 2 months after the admission.

**Lessons:**

NF can occur during the perioperative period for routine clean radius plate removal operation in patients with no risk factor for NF. The objective is to remind the physicians to stay aware of this disease, especially its early clinical signs and symptoms. Urgent subsequent treatment, including surgical debridement, antibiotic therapy, and supporting management, is the key to ensure the survival and better prognosis of patients.

## 1 Introduction

The term “necrotizing fasciitis” (NF) was created by Wilson in 1952^[1]^ for a rare infection characterized by a rapidly progressive and widespread necrosis of the skin, subcutaneous tissue, and superficial fascia.^[2]^ Despite improved diagnostic tools and management of treatment in recent years, NF still has a high mortality rate ranging from 6% to 76%.^[2]^ Recognized risk factors include age >60 years, diabetes, obesity, immunosuppression, and malnutrition.^[3]^ Many studies have associated the cause of NF with penetrating injury, open trauma, burns, etc.^[4]^ However, the development of NF in patients with no risk factor and from a clean minor surgery was a rare occurrence. An early diagnosis and urgent subsequent treatment of NF is the key to ensure the safety of a patient's life, since a delayed recognition can lead to a rapid progression and even catastrophic systemic inflammatory response syndrome.^[3]^ But to date, no definite diagnostic criteria is available for NF. Early-stage NF can be challenging for physicians due to a lack of typical cutaneous features and sometimes deceiving laboratory findings, and are therefore frequently misdiagnosed as cellulitis or abscess.^[5]^

This study describes a successfully treated case of NF after a routine clean surgery of right distal radius bone plate removal. The extensiveness of NF's rapid progression in the present case has perhaps not been previously reported. The present study aims to emphasize the possible risk of NF after a minor sterile surgery and highlight that physicians must stay vigilant to make early diagnosis and timely treatment of this disease to ensure a successful outcome. This study discusses and summarizes the clinical experience in diagnosing and treating NF, supplemented by current concept and management in previous studies. This case report was approved by the ethics committee of our hospital (Institutional Review Board of Shanghai 9th Peoples Hospital Affiliated to Shanghai Jiao Tong University School of Medicine). The patient agreed with potential publication of his case and signed the patient consent.

## 2 Case report

**Figure 1:**
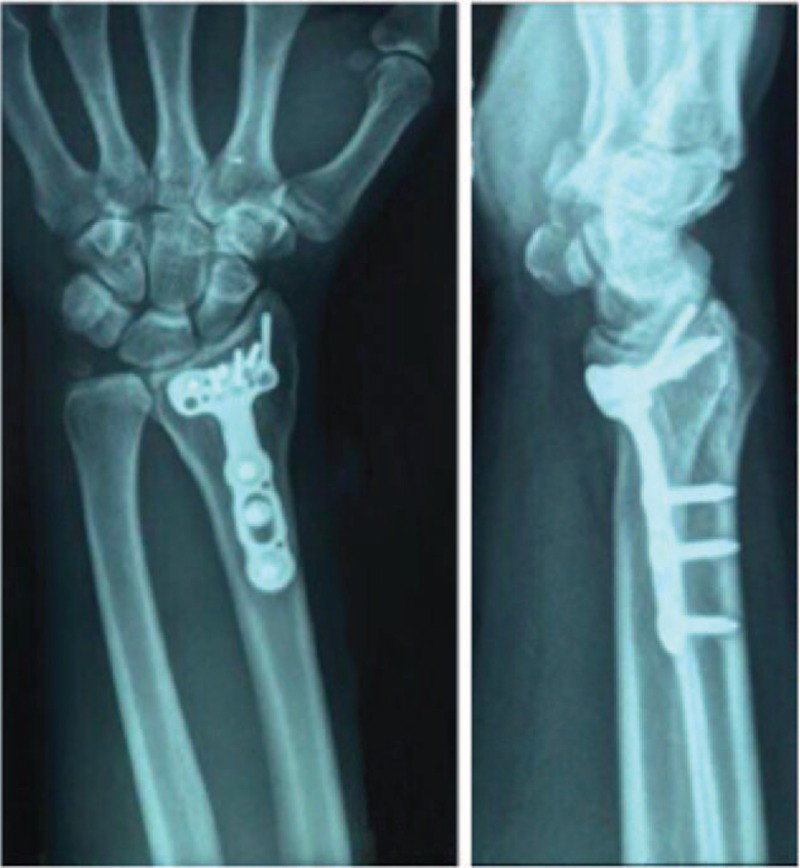
Preoperative x-ray of right radius plate removal surgery.

**Figure 2:**
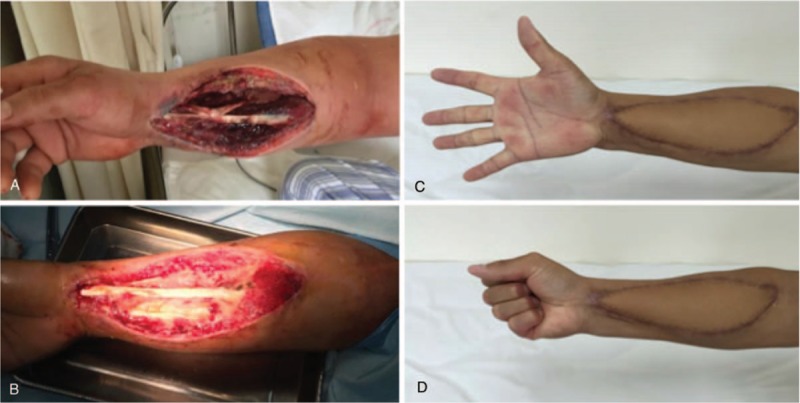
A, Right forearm wound after incision and decompression in treating compartment syndrome (on admission, 3rd day). B, Right forearm wound on the 34th day of the disease, with the wound surface covered by pedicled skin flap later on. C and D, Right forearm 6 months after skin graft, with no hand inactivity or rigidity.

A 35-year-old man, who had no comorbidity and experienced no discomfort, underwent a clean removal of right distal radius bone plate for his personal demand in a regional hospital 66 days ago (Fig. 1), which was planted for his right radius fracture 3 years ago. Preventive antibiotic drug was used preoperatively and no nonsteroidal anti-inflammatory drug (NSAID) was used postoperatively. However, 12 hours after the surgery, the patient claimed numbness and intense pain (visual analogue scale [VAS] 9–10), with redness and swelling observed in the operation area. His vital signs were normal. He was diagnosed with compartment syndrome and underwent incision and decompression of his right forearm within 24 hours after the initial surgery (Fig. 2A). During the surgery, few limpid liquids were observed subcutaneously, and the anterior muscular group was exposed, with no necrotizing tissue found. However, the patient did not get any relief from pain in his right forearm, and the swelling extended to his right arm. He was therefore transferred to the emergency department of our hospital on the third postoperative day.

**Figure 3:**
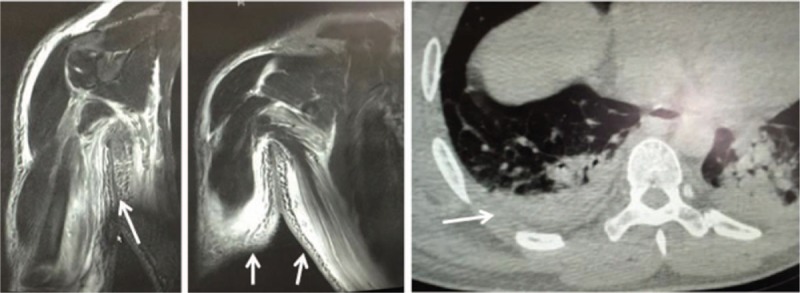
Magnetic resonance imaging (MRI) and computerized tomography (CT) displayed obvious soft tissue swelling of right forearm and thoracic walls (arrow).

On admission, his vital signs were a temperature of 37°C, pulse of 120 beats/min, respiratory rate of 22 times/min, and blood pressure of 130/70 mm Hg. His physical examination revealed a large surgical cut on the volar side by previous incision and decompression, exposing a slightly dark-colored anterior muscular group of forearms (Fig. 2A). His right radius fingers presented passive dragalgia, with a palpable right radius artery. His right upper arm had swelling and redness but without pressing pain. The laboratory examination revealed an abnormal white blood cell count (WCC) of 1.9 × 10^9^/L, platelet (PLT) of 36 × 10^9^/L, activated partial thromboplastin time of 57.2 s, and an increased myohemoglobin of 528.0 ng/mL. As the C-reactive protein (CRP) level was not tested at that time, the laboratory risk indicator for necrotizing fasciitis (LRINEC) score was assumed to be around 5, indicative of potential NF. Computerized tomography (CT) displayed swelling of soft tissue in right thoracic walls and fluid collection in the muscle space (Fig. 3). Compartment syndrome was excluded. Initial diagnoses were skin infection such as cellulitis and abscess, whereas NF was also suspected, and the patient received an empirical antibiotic therapy of intravenous cefonicid sodium (2 g BID for 3 days). NSAID was added but his pain relief is only temporary. Other supporting management included treatment to prevent septic shock, anemia, metabolic acidosis, electrolyte imbalance, and multiple organ failure.

On the fourth day, surgical debridement was performed to remove the obviously swollen tissue in his right forearm under local anesthesia, with vacuum sealing drainage (VSD) applied to the wound surface to help drain the fluid. During the operation, few light-colored liquids were observed, without pus. The fascia was noted to be slightly grayish. These finding suggested fasciitis, whereas cellulitis was less likely. Postoperatively, the patient's condition continued to deteriorate. The swelling and redness extended to his right neck, thorax, and dorsum, accompanied with prominent local tension blisters. Laboratory tests exhibited continuous declination of the PLT and increasing inflammatory markers. His LRINEC score was 2.

On the fifth day, the swelling continued to extend. Laboratory examinations exhibited a further declined PLT and increased inflammatory markers. His LRINEC score was 2, indicating a lesser possibility for NF. Serum lactate was tested 4.9 mmol/L, showing a strong association with NF (>2.0 mmol/L). The magnetic resonance imaging (MRI) displayed obvious soft tissue swelling in the right forearm and thoracic walls, as well as emphysema in the neck and mediastinum (Fig. 3). Group consultation of multisector doctors concluded a high possibility of NF, based on the evidences so far: clinical manifestations including alarming sign of disproportionately strong pain, local tension blisters, and rapid progression; laboratory examinations including an increased serum lactate despite a lesser LRINEC score; and CT and MRI showing soft tissue swelling. The antibiotic drug was changed to vancomycin (1 g TID) and meropenem (1 g BID), with the use of intravenous gamma globulin (10 g QD for 2 day) and methylprednisolone hormone (80 mg QD for 3 days). An incision and subsequent decompression were performed in the area of the right neck, subclavius, thoracic walls, and hypochondrium. During the operation, limpid and light-colored yellow liquid between fascia and subcutaneous tissue was observed, without obvious gas, pus, or necrotizing tissue. The catheter was retained in place for head and neck drainage and VSD for other regions. The patient was transferred to the surgical intensive care unit (SICU) postoperatively for a 24-hour monitoring. The follow-up debridement was well prepared.

**Figure 4:**
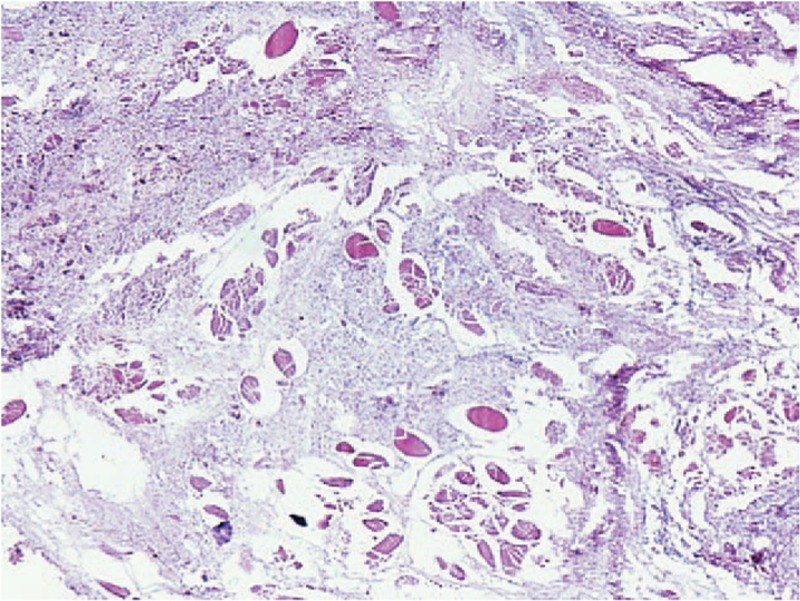
Histological examination of the right forearm wound (hematoxylin and eosin staining, ×100).

On the sixth day, the cutaneous swelling appeared in the head and neck despite incision and decompression performed on the previous day. He experienced intense pain (VAS 8–10) in the region, a hoarse throat, and dyspnea, with difficulty in opening his eyes and mouth. An urgent surgical debridement was performed in the infraorbital, buccal, and submental space areas, along with tracheotomy to prevent apnea. Postoperatively, the swelling stopped expanding and the patient's condition gradually improved and stabilized. Previous wound bacterial culture from the first debridement on the fourth day exhibited gram-positive *Streptococcus pyogenes*, which was sensitive to methicillin, vancomycin, and meropenem. Meropenem usage maintained the same, whereas vancomycin was changed to 1 g BID since the patient's condition improved. Both antibiotic drugs were used until the 39th day (for 35 days). Also, the histological examination result of the right forearm confirmed NF, which exhibited necrotizing subcutaneous tissue with inflammatory leukocyte infiltration (Fig. 4).

**Figure 5:**
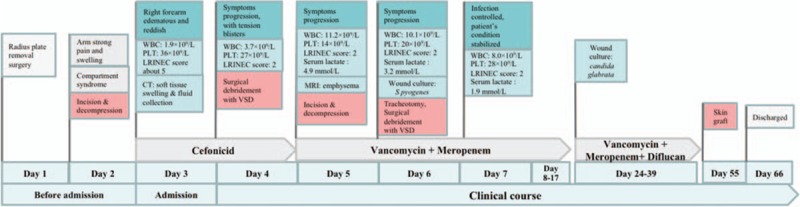
Brief clinical course and treatment strategy of the patient.

Several other local debridements were performed from the 14th day, and new wound culture revealed fungal infection caused by *Candida glabrata*; therefore, antifungal drug fluconazole (Diflucan; 200 mg QD for 16 days) was added according to the antibiotic sensitivity test on the 24th day. On the 52nd day, the wounds in maxillofacial region and thoracic walls were healed. The wound in the right forearm was dry with healthy granulation (Fig. 2B). On the 55th day, free pedicled skin flap graft from his left anterolateral thigh was performed in his right forearm. He was discharged from the hospital on the 66th day. He has been followed up every 3 to 6 months (Fig. 2C and D). Currently, his right forearm functions normally, with no hand inactivity or rigidity. Figure 5 summarizes the clinical course of this disease.

## 3 Discussion

Early-stage NF lacks typical cutaneous features, which can often be deceitful for physicians to realize the severity of subcutaneous infection. Several signs and symptoms are indicative of NF diagnosis, including disproportionately strong pain, extreme inflammation, and ecchymosis. Disproportionate pain is presented in almost every case of NF.^[2,6]^ However, as NSAID is often prescribed for patients in the early stage, the true level of pain can be masked. The patient in the present study presented an intense pain (VAS 9–10), out of proportion of his original physical findings, due to the fact that he received only preoperative antibiotics after the initial radius plate removal surgery, without pain-relief drugs in the early phases of his hospitalization (day 1–3). It can be educational for orthopedic surgeons to consider carefully the use of NSAID in similar circumstances. The LRINEC score is currently an acknowledged tool for early diagnosis, which was designed by Wong et al^[7]^ in 2004 based on laboratory tests including patient's WCC, CRP, hemoglobin, serum glucose, creatinine, and sodium. However, the LRINEC score of the patient in the present study remained around or below 5 all along the course of NF, indicating a possibility of NF lesser than 50%. Murphy et al^[8]^ described that serum lactate of more than 2.0 mmol/L is strongly associated with NF, which is in accordance with the present case. Therefore, for early-stage NF, orthopedic surgeons should form a right and clear suspicion from a paucity of clinical evidence, supplemented with different laboratory findings. Imaging techniques, histological examinations, and wound culture are relatively time consuming and are not suggested for an early diagnosis.

Surgical debridement to excise subcutaneous necrotic tissue should be performed as soon as NF is suspected. Delayed surgical debridement can definitely increase patient mortality.^[9]^ As NF is rapidly progressive, serial debridement can be performed within the first 24 hours after the initial debridement, and a follow-up debridement should always be prepared, as performed in SICU in the present case. Tracheotomy is only necessary when airway gets involved. As for antibiotics, a wide-spectrum coverage with 3 antibiotics is recommended, covering gram-positive, gram-negative, and anaerobic bacteria.^[10]^ However, at an early stage until blood culture results are available, a combination of 2 antibiotics can fulfill enough coverage, such as vancomycin and meropenem in the present case. Antibiotic therapy should be adjusted in response to wound culture results. In the present case, although an early suspicion of NF was made on the third day, a wider coverage of antibiotics was prescribed not until the fifth day when more evidences of NF appeared. This may the cause of its rapid progression in the early stage despites prompt surgical interventions. Apart from surgical debridement and antibiotic therapies, other interventions were implemented in the patient, such as transference to SICU, tracheotomy before the extension of the swelling to the airway, and supporting treatment and postoperative wound repair using skin graft.

In summary, an early diagnosis of NF is relied on the awareness of the physicians about this disease, especially its clinical signs and symptoms. Urgent subsequent treatment of NF, including surgical debridement, antibiotic therapy, and supporting management, is the key to ensure the survival and better prognosis of patients.

## Author contributions

**Writing – original draft:** Yuchen Cai.

**Writing – review & editing:** Yuchen Cai, Yaokai Gan, Chao Yu, Jian Tang, Yuehua Sun.
